# Exploring the nonlinear relationship between serum uric acid to high-density lipoprotein cholesterol ratio and obesity in older adults: a cross-sectional study

**DOI:** 10.3389/fpubh.2025.1587194

**Published:** 2025-05-01

**Authors:** Fanchang Wang, Hongyang Qiao, Yi Zheng, Yating Zheng, Yuxin Ni, Xiaoming He

**Affiliations:** ^1^The Second Clinical Medical College of Zhejiang Chinese Medical University, Hangzhou, China; ^2^The First Affiliated Hospital of Wenzhou Medical University, Wenzhou, China; ^3^The Second Affiliated Hospital of Zhejiang Chinese Medical University, Hangzhou, China

**Keywords:** uric acid to high-density lipoprotein cholesterol ratio (UHR), HDL cholesterol, uric acid, geriatric obesity, NHANES, metabolic syndrome

## Abstract

**Background:**

The prevalence of obesity, a common metabolic disorder, has been increasing annually, particularly in older adults. This trend poses a significant socioeconomic burden. The uric acid to high-density lipoprotein cholesterol ratio (UHR) was defined by dividing UA (mg/dL) by HDL-C (mg/dL) and multiplying by 100%. According to recent clinical research, UHR has emerged as a potential innovative indicator in metabolic status evaluation, supported by contemporary biomarker research. This cross-sectional study investigated the association between the UHR index and obesity prevalence among older Americans.

**Objective:**

This cross-sectional research employed nationally representative survey data to Examine the connection between the UHR index and obesity among older individuals aged 60 and above.

**Methods:**

This study utilized data from the National Health and Nutrition Examination Survey (NHANES) spanning 2011 to 2016. Individuals who were 60 years old or older were included in the study (*n* = 3,822). The relationship between UHR levels and obesity (as measured by a body mass index of 30 kg/m^2^ or greater or a waist-to-height ratio (WHtR) ≥0.5) was investigated using weighted multivariable logistic regression analyses, with adjustments made for sociodemographic characteristics, behavioral patterns, and clinical covariates, adjusting for sociodemographic, behavioral, and clinical covariates. Restricted cubic spline, ROC curves, threshold analysis, and subgroup analysis were also used.

**Result:**

After full adjustment for confounders, UHR was positively associated with the risk of obesity as defined by BMI (highest quartile vs. lowest quartile: OR = 6.13, 95% CI = 4.01–9.39; *P*-trend < 0.001) and UHR was positively associated with the risk of obesity as defined by WHtR (highest quartile vs. lowest quartile: OR = 20.21, 95% CI = 8.33–49.02; *p*-trend < 0.001). In addition, The restricted cubic spline analysis uncovered a nonlinear dose-response relationship (P < 0.01), and threshold analysis found inflection points of −2.485 in obesity defined by BMI and −2.503 in WHtR. Subgroup analyses showed that the association between UHR and obesity in older Americans was consistent across subgroups, demonstrating high reliability (all P-interaction > 0.05). The AUC for UHR predicting obesity defined by BMI was calculated to be 0.65 (95% CI = 0.63–0.66). The UHR predicted AUC for obesity as defined by men's body mass index (BMI) was 0.67 (95% CI = 0.65–0.70). UHR predicted an AUC of 0.69 (95% CI = 0.67–0.72) for obesity defined by body mass index (BMI) in females. The AUC for UHR predicting obesity defined by WHtR was calculated to be 0.75 (95% CI = 0.72–0.78). UHR predicted an AUC of 0.76 (95% CI = 0.72–0.80) for obesity defined by WHtR in males, and UHR predicted an AUC of 0.83 (95% CI = 0.79–0.87) for obesity defined by WHtR in females.

**Conclusion:**

The findings demonstrate a notable positive correlation between UHR and obesity in older adults, with this association remaining evident following adjustment for multiple confounding variables. These results imply that systematic evaluation of UHR levels could serve as an effective strategy for proactively detecting populations susceptible to obesity-related metabolic disorders.

## Introduction

Obesity, a prevalent clinical metabolic disorder, is conventionally defined as a Body Mass Index (BMI) ≥30.0 (1) or waist-to-height ratio (WHtR) ≥ 0.5 (2). Recent epidemiological data indicate that 38% of the global population is classified as overweight or obese, with projections estimating a rise to 51% by 2035 (3). Concurrently, the aging global population has been associated with a rising prevalence of obesity among older adults (4). Obesity in older adults is strongly linked to severe comorbidities, including cardiovascular disease (5), type 2 diabetes mellitus (6), hypertension (7), and dyslipidemia (8), thereby imposing substantial socioeconomic burdens and contributing to escalating global mortality rates (9). Traditional modalities for obesity assessment, such as Magnetic resonance imaging (MRI), are often technically complex and financially prohibitive, limiting their utility in clinical practice and large-scale epidemiological studies (10). Furthermore, the association between BMI and obesity is influenced by demographic variables such as race, gender, and age (11). Critically, BMI fails to adequately capture the pathophysiological impact of excess adiposity, potentially leading to underestimation of obesity-related health risks (12). Epidemiologic studies investigating obesity in older adults have demonstrated that BMI has less ability than waist-to-height ratio (WHtR) to distinguish individuals with high muscle mass from those with excess fat or abdominal obesity (13). UHR can be calculated from routine blood tests, thus avoiding measurement errors common in anthropometric indices (e.g., posture-related waist-to-height ratios). This is particularly advantageous for bedridden or frail older adults for whom accurate anthropometric measurements are difficult. UHR is less expensive than MRI and has a shorter detection time. More importantly, UHR does not require specialized operators. Therefore, to determine the relationship between UHR and obesity in older adults and to identify a simple and convenient health assessment metric, we assessed cross-sectional associations between UHR and obesity in older adults as defined by BMI and obesity in older adults as defined by WHtR using data from the National Health and Nutrition Examination Survey (NHANES) conducted in the United States from 2011 to 2016.

The mechanistic interplay between composite metabolic indices and obesity in older adults has garnered significant research interest In the last few years (14). Uric acid (UA), the terminal product of purine metabolism, exhibits dual roles in energy homeostasis. On the one hand, UA potentiates lipid accumulation via activating adipogenesis-related enzymes (15); conversely, its antioxidative properties may mitigate metabolic dysregulation (16). In contrast, high-density lipoprotein cholesterol (HDL-C) is essential for maintaining cholesterol homeostasis and modulating lipid metabolism through its mediation of reverse cholesterol transport (17). The uric acid to HDL-C ratio (UHR) is particularly important in older adults due to age-related physiological and socio-environmental vulnerabilities. Aging is characterized by a progressive decrease in renal uric acid excretion and HDL cholesterol synthesis (18, 19), thus creating a metabolic environment conducive to an elevated UHR. Mechanistically, elevated UHR exacerbates oxidative stress and adipogenesis, thereby promoting obesity. Hyperuricemia, a key component of UHR, is associated with increased reactive oxygen species (ROS) production (20), which impairs mitochondrial function in adipocytes, accelerating lipid peroxidation and ectopic fat deposition (21). At the same time, low HDL-C levels impair cholesterol reverse transporter and antioxidant defenses, further amplifying oxidative damage and adipose tissue inflammation (22). These processes may synergistically drive adipocyte hypertrophy and visceral fat accumulation, leading to metabolic imbalances. In turn, oxidative stress and chronic inflammation are central to the pathogenesis of aging-related diseases such as cardiovascular and kidney diseases (23, 24). Studies have shown that elevated UHR is inversely correlated with circulating alpha-klotho, suggesting that it has utility in predicting renal aging and frailty (25). Long-term cross-sectional studies also support this hypothesis. For example, it has been shown that severe obesity is strongly associated with elevated serum uric acid and lowered high-density lipoprotein cholesterol (26, 27). While UA levels demonstrate a J-curve association with obesity (28), reduced HDL-C is strongly linked to central adiposity (29). Although obesity-related indicators such as body mass index (BMI) and waist circumference are closely associated with physiological aging, substantial inter-individual variability exists in the predictive performance of these biomarkers in older adults (30). Emerging studies indicate that UHR is pivotal to age-related body composition changes (25, 31). As a composite metric, UHR sensitively reflects dysregulation of the lipid-purine metabolic axis, with elevated values demonstrating robust correlations with visceral fat accumulation and insulin resistance (32). Nevertheless, the clinical utility of UHR in geriatric obesity assessment remains underexplored, particularly given age-specific physiological shifts such as dynamic muscle-to-fat ratio alterations and attenuated metabolic compensatory mechanisms. Therefore, elucidating the relationship between UHR and obesity in aging populations may yield a novel biomarker for the early detection of geriatric obesity.

The well-established association between the urea-to-creatinine ratio (UHR) and inflammatory pathologies, including hypertension (33) and non-alcoholic fatty liver disease (NAFLD) (34), prompts scientific inquiry into its potential role in geriatric obesity. Current evidence remains scarce regarding UHR's specific relationship with obesity in the aging population. Therefore, we aimed to elucidate the relationship between UHR and obesity in older Americans.

## Materials and methods

NHANES employs a complex probabilistic sampling methodology across multiple stages to generate nationally representative estimates through its population-based cross-sectional design (35). Given NHANES's complex multistage sampling design, we used survey weights to ensure that the estimates were nationally representative. Ethics clearance was obtained from the National Center for Health Statistics Institutional Review Board prior to study implementation, with documented informed consent acquired from all subjects preceding any research procedures.

### Study population

**Figure 1:**
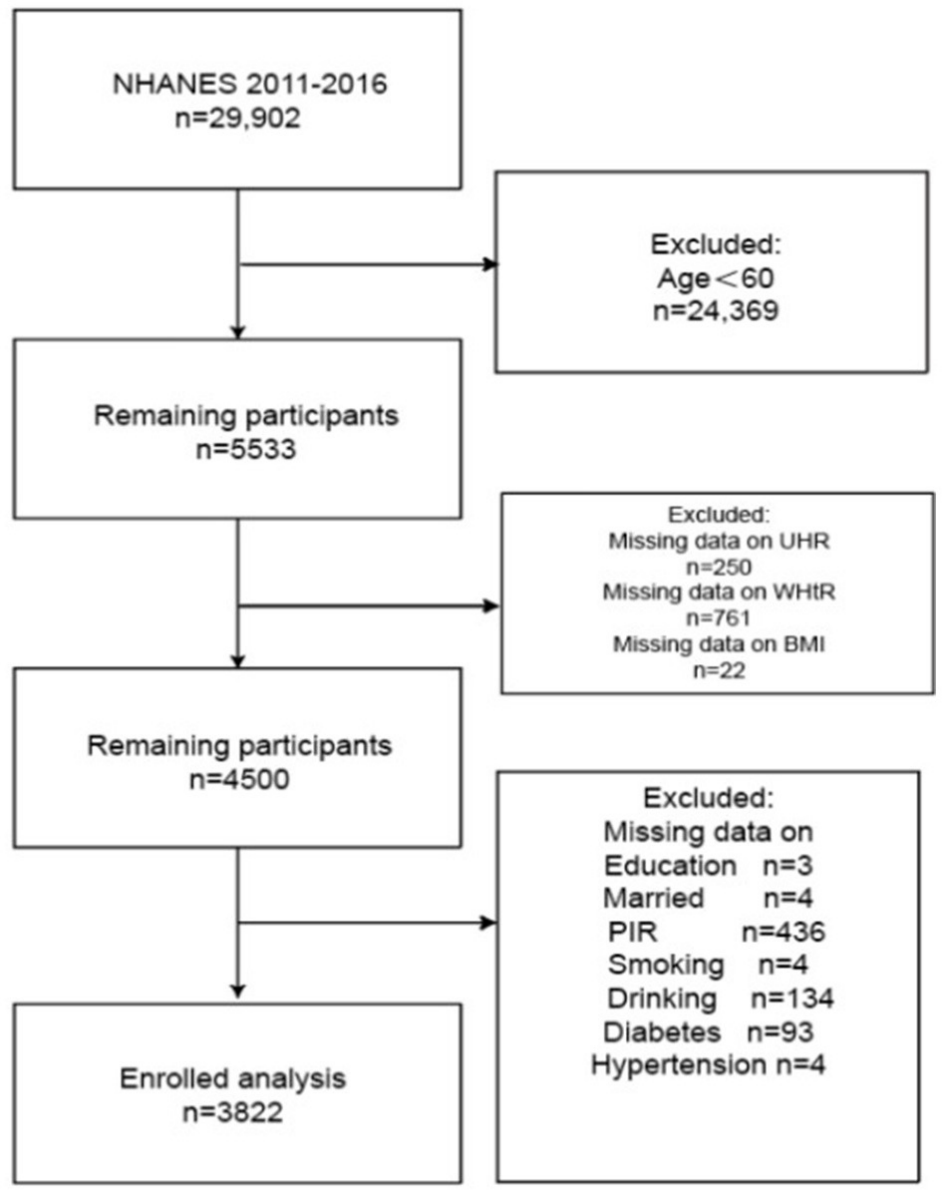
Flow-chart of the study samples.

We analyzed pooled data from three NHANES cycles (2011–2016). We handle missing data by deleting column by column. From an initial sample of 29,902 participants, exclusion criteria were systematically applied: (1) participants younger than 60 years of age (*n* = 24,369); (2) 22 subjects lacked BMI measurements, 250 lacked the UHR index, and 761 lacked the WHtR; subsequent exclusions included those with missing data on educational attainment (*n* = 3), Poverty Income Ratio (PIR, *n* = 436), alcohol consumption (*n* = 134), smoking status (*n* = 4), marital status (*n* = 4), diabetes (*n* = 93), and hypertension (*n* = 4). The final study population consisted of 3,822 eligible participants (Figure 1).

### Definitions of exposure and outcome variables

Following the National Heart, Lung, and Blood Institute (NHLBI) criteria, obesity among older adults was defined, and body mass index (BMI) was calculated by dividing weight in kilograms by height in meters squared (kg/m^2^). Participants were then classified into obese (BMI ≥ 30) or non-obese (BMI < 30) groups (36). To account for the limitations of the older population, where changes in body composition and sarcopenia may confound BMI, we introduced an alternative way of defining obesity, namely waist-to-height ratio (WHtR) ≥ 0.5 (2).

The uric acid to high-density lipoprotein cholesterol ratio (UHR) served as the exposure variable in this research. The Uric acid-to-HDL ratio (UHR) was defined by dividing UA (mg/dL) by HDL-C (mg/dL) and multiplying by 100%. The data necessary for calculating the UHR were derived from the physical examination records of the National Health and Nutrition Examination Survey (NHANES) spanning the years 2011–2016. The UHR consists of UA and HDL-C, measured by blood in the morning in a fasting state, with the subject fasting for 8–12 h before blood collection. UA is measured: Serum uric acid concentration is measured by the DxC800 Automated Laboratory Analyzer using the timed endpoint method, where the enzyme urease reacts with uric acid to produce hydrogen peroxide. Peroxidase promotes the reaction of aminoantipyrine (4-AAP) and 3,5-dichloro-2-hydroxybenzenesulfonate (DCHBS) with hydrogen peroxide, and the resulting product is measured at 520 nm. HDL-C is measured by adding magnesium sulfate solution to the sample to form a complex with the non-HDL-C, with the aim of not subsequently reacting with the measurement reagent. The next step is converting HDL cholesterol esters to HDL cholesterol by polyethylene glycol esterase. Finally, 4-amino antipyrine and HSDA react with the hydrogen peroxide generated from the reaction to form a blue dye, and finally, HDL-C levels are determined by photometric measurement at 600 nm using a Hitachi 7600 fully automated biochemical analyzer (25). UHR values (expressed in mg/dL for both components) were categorized into quartiles (Q1-Q4) to ensure the accuracy of the data (37).

### Covariates

For smoking history, we based the question, “Have you ever smoked at least 100 cigarettes in your life?” We categorized them as having a history of smoking and not having a history of smoking. High-risk alcohol consumption was identified as consuming four or more drinks per day (38). Physical activity levels were divided into low (< 500 MET min/week) and high (≥500 MET min/week) categories, in line with national guidelines (39). Clinical characteristics were determined through standardized measurements and validated criteria. Diagnostic criteria for hypertension included fulfillment of at least one of these parameters: (1) recorded systolic blood pressure ≥140 mmHg; (2) measured diastolic blood pressure ≥ 90 mmHg; (3) physician-confirmed medical history disclosed by participants; or (4) active pharmacological treatment for blood pressure regulation (40). Identification of type 2 diabetes mellitus (T2DM) required meeting any of these established indicators: (1) medically verified patient disclosure; (2) prescribed administration of antidiabetic medications; (3) fasting blood glucose levels ≥126 mg/dL (7.0 mmol/L); or (4) hemoglobin A1c values ≥6.5% (41). Comprehensive operational definitions for all study variables are documented in the standardized National Health and Nutrition Examination Survey (NHANES) methodological guidelines, accessible at: https://www.cdc.gov/nchs/nhanes/.

### Statistical analysis

Statistical analyses were performed using R statistical software (version 4.3.1). The significance of all statistical tests was *p* < 0.05. The National Health and Nutrition Examination Survey (NHANES) employed a multistage, stratified sampling approach to collect nationally representative data, incorporating appropriate sample weights to ensure population representativeness. By NHANES analytical guidelines, sample weights were applied throughout our analyses to account for the complex survey design. Since the heterogeneous distribution of UHR data may affect model stability, we log-transform the data to improve model stability. Weighted multivariate logistic regression analyses were conducted to evaluate the association between the UHR index and obesity risk in older adults. The UHR index was categorized into quartiles (Q1–Q4), with the lowest quartile (Q1) as the reference category. Three distinct models were constructed: Model 1 presented crude estimates without covariate adjustment, while subsequent models progressively adjusted for demographic characteristics (age, sex, race), socioeconomic factors (education level, marital status, poverty-income ratio), lifestyle variables (smoking status, alcohol consumption, physical activity equivalents), and clinical comorbidities (hypertension, diabetes mellitus). Post-adjustment for covariates, Smooth curve fitting was performed to examine the association between UHR and obesity in older adults, threshold effects for nonlinear models were determined, and meaningful inflection points were identified. In addition, subgroup analyses were conducted across demographic characteristics, behavioral patterns, and health status, and interaction terms were added to test for heterogeneity between subgroups. All results are reported as odds ratios (ORs) with corresponding 95% confidence intervals (CIs) and p-values. Finally, the predictive performance of the UHR for obesity in older adults, as well as older men and women, was assessed using the subject's work characteristic curve (ROC). We also determined the critical value of the UHR through ROC analysis.

## Results

### Baseline characteristics

The study involved 3,822 subjects comprising 1,900 males and 1,922 females. Table 1 shows that out of 3,822 subjects, 1,466 (38.36%) were diagnosed as obese. The obese group had a significantly higher UHR and a younger mean age compared to the non-obese group. Slightly lower economic levels in the obese group relative to the non-obese group and there were significant differences in racial distribution: higher proportions of non-Hispanic whites and non-Hispanic blacks and lower proportions of other races (including multiracial). In terms of educational attainment, those with some college or AA degree were more highly represented among the obese. Marital status showed that a higher proportion of the obese group was married. Behavioral characteristics showed that the obese group had a higher proportion of smokers, a higher proportion of alcohol drinkers, and a significantly higher proportion of high-intensity exercisers. In terms of health status, the frequency of hypertension was much higher in the obese group than in the non-obese group (*p* < 0.001 in all cases), and the frequency of diabetes mellitus in the obese group was similar to the frequency of non-diabetes mellitus. In conclusion, there were significant differences between the obese and non-obese groups in terms of age, UHR, WHtR, PIR, race, literacy, marital status, smoking history, physical activity, and health status (hypertension and diabetes) (*p* < 0.001).

**Table 1:** Baseline characteristics of the study participants.

**Variable**	**Total (*n* = 3,822)**	**Non-obesity (*n* = 2,356)**	**Obesity (*n* = 1,466)**	***P***
Age	69.14 (0.17)	69.80 (0.25)	68.10 (0.23)	**< 0.001**
PIR	3.09 (0.07)	3.16 (0.08)	2.99 (0.10)	**0.045**
Gender				0.121
Male	1,900 (46.45)	1,250 (47.81)	650 (44.31)	
Female	1,922 (53.55)	1,106 (52.19)	816 (55.69)	
Race				**< 0.001**
Mexican American	451 (3.86)	237 (3.31)	214 (4.73)	
Other Hispanic	449 (3.65)	273 (3.70)	176 (3.58)	
Non-Hispanic White	1,748 (78.85)	1,097 (79.01)	651 (78.59)	
Non-Hispanic Black	799 (7.81)	436 (6.87)	363 (9.28)	
Other race—including multiracial	375 (5.84)	313 (7.12)	62 (3.83)	
Education				**< 0.001**
< 9th grade	548 (6.71)	329 (6.89)	219 (6.44)	
9–11th grade	487 (9.24)	292 (9.05)	195 (9.52)	
High school grad/GED or equivalent	888 (22.49)	526 (21.18)	362 (24.56)	
Some college or AA degree	1,064 (31.83)	624 (29.50)	440 (35.49)	
College graduate or above	835 (29.73)	585 (33.38)	250 (23.99)	
Married				**0.030**
Yes	2,119 (62.37)	1,330 (64.54)	789 (58.97)	
No	1,703 (37.63)	1,026 (35.46)	677 (41.03)	
Smoking				**0.032**
Yes	1,934 (51.23)	1,199 (49.12)	735 (54.55)	
No	1,888 (48.77)	1,157 (50.88)	731 (45.45)	
Alcohol				0.102
Yes	2,546 (72.39)	1,613 (73.54)	933 (70.58)	
No	1,276 (27.61)	743 (26.46)	533 (29.42)	
Physical activity				**< 0.001**
High physical activity	1,905 (45.66)	1,085 (40.80)	820 (53.27)	
Low physical activity	1,917 (54.34)	1,271 (59.20)	646 (46.73)	
Hypertension				**< 0.001**
Yes	2,686 (66.45)	1,557 (61.11)	1,129 (74.81)	
No	1,136 (33.55)	799 (38.89)	337 (25.19)	
Diabetes				**< 0.001**
Yes	1,354 (28.74)	660 (20.39)	694 (41.84)	
No	2,468 (71.26)	1,696 (79.61)	772 (58.16)	
UHR	0.11 (0.00)	0.10 (0.00)	0.13 (0.00)	**< 0.001**
WHtR	0.62 (0.00)	0.57 (0.00)	0.72 (0.00)	**< 0.001**

UHR, UA (mg/dL) by HDL-C (mg/dL) and multiplying by 100%.

WHtR, waist-to-height ratio.

Bolded *p*-values: statistically significant.

Table 2 summarizes the baseline characteristics of older adults stratified by quartiles of the UHR index to investigate the potential association between UHR levels and the incidence of obesity in older adults (Table 2). The UHR index was categorized into four quartiles, i.e., Q1, Q2, Q3, and Q4. Those in the fourth quartile had a higher body mass index (BMI), a lower level of household income, and a higher prevalence of smoking. The BMI, PIR, WHtR, gender, ethnicity, education, marital status, smoking status, and physical activity prevalence of hypertension, diabetes, and obesity significantly differed between the first, second, third, and fourth quarters (*p* < 0.05). The prevalence of obesity increased progressively with increasing quartiles of the UHR index (*P*-trend < 0.001).

**Table 2:** Baseline characteristics according to UHR index qualities.

**Variable**	**Total (*n* = 3,822)**	**Q1 (*n* = 831)**	**Q2 (*n* = 955)**	**Q3 (*n* = 1,024)**	**Q4 (*n* = 1,012)**	***P***
BMI	29.23 (0.19)	25.99 (0.37)	28.99 (0.28)	30.20 (0.30)	31.73 (0.34)	**< 0.001**
Age	69.14 (0.17)	69.13 (0.34)	69.40 (0.26)	69.09 (0.34)	68.93 (0.24)	0.410
PIR	3.09 (0.07)	3.33 (0.12)	3.12 (0.09)	2.94 (0.09)	2.98 (0.08)	**< 0.001**
WHtR	0.62 (0.00)	0.57 (0.01)	0.62 (0.00)	0.64 (0.00)	0.65 (0.00)	**< 0.001**
Gender						**< 0.001**
Male	1,900 (46.45)	191 (20.15)	391 (38.49)	570 (54.94)	748 (72.15)	
Female	1,922 (53.55)	640 (79.85)	564 (61.51)	454 (45.06)	264 (27.85)	
Race						**0.002**
Mexican American	451 (3.86)	84 (2.98)	112 (3.91)	142 (4.83)	113 (3.73)	
Other Hispanic	449 (3.65)	84 (2.52)	130 (4.57)	112 (3.59)	123 (3.91)	
Non-Hispanic White	1,748 (78.85)	405 (82.41)	432 (78.49)	443 (76.38)	468 (78.11)	
Non-Hispanic Black	799 (7.81)	175 (7.46)	193 (7.77)	224 (8.65)	207 (7.34)	
Other race—including multiracial	375 (5.84)	83 (4.63)	88 (5.25)	103 (6.55)	101 (6.91)	
Education						**< 0.001**
< 9th grade	548 (6.71)	95 (4.31)	146 (7.45)	150 (7.35)	157 (7.74)	
9–11th Grade	487 (9.24)	95 (7.25)	118 (9.06)	132 (10.64)	142 (9.99)	
High school grad/GED or equivalent	888 (22.49)	177 (21.92)	205 (20.63)	245 (22.91)	261 (24.52)	
Some college or AA degree	1,064 (31.83)	240 (30.23)	255 (29.35)	307 (34.68)	262 (33.07)	
College graduate or above	835 (29.73)	224 (36.29)	231 (33.51)	190 (24.42)	190 (24.68)	
Married						**0.014**
Yes	2,119 (62.37)	428 (61.99)	482 (57.26)	598 (63.64)	611 (66.58)	
No	1,703 (37.63)	403 (38.01)	473 (42.74)	426 (36.36)	401 (33.42)	
Smoking						**< 0.001**
Yes	1,934 (51.23)	348 (45.32)	443 (46.42)	545 (52.66)	598 (60.52)	
No	1,888 (48.77)	483 (54.68)	512 (53.58)	479 (47.34)	414 (39.48)	
Alcohol						0.316
Yes	2,546 (72.39)	534 (72.04)	608 (69.73)	687 (72.69)	717 (75.10)	
No	1,276 (27.61)	297 (27.96)	347 (30.27)	337 (27.31)	295 (24.90)	
Physical activity						**< 0.001**
High physical activity	1,905 (45.66)	404 (42.01)	434 (40.77)	526 (49.57)	541 (50.29)	
Low physical activity	1,917 (54.34)	427 (57.99)	521 (59.23)	498 (50.43)	471 (49.71)	
Hypertension						**< 0.001**
Yes	2,686 (66.45)	519 (56.88)	661 (62.97)	751 (71.10)	755 (74.81)	
No	1,136 (33.55)	312 (43.12)	294 (37.03)	273 (28.90)	257 (25.19)	
Diabetes						**< 0.001**
Yes	1,354 (28.74)	177 (14.41)	300 (23.49)	378 (31.64)	499 (45.39)	
No	2,468 (71.26)	654 (85.59)	655 (76.51)	646 (68.36)	513 (54.61)	

Bolded *p*-values: statistically significant.

### Correlation between UHR index and obesity in older adults

To assess the association between UHR and obesity risk in older adults, we used a multivariate logistic regression model. Because the heterogeneous distribution of UHR data may affect model stability, we log-transformed the UHR data, and the unadjusted and adjusted models are shown in Tables 3, 4. For the association between UHR and obesity in older adults defined by BMI, as shown in Model 1, without any adjustment for variables, the odds ratio (OR) was 4.25 (95% CI, 3.39–5.32; *P* < 0.001). The odds ratio (OR) was 6.56 (95% CI, 4.92–8.75; *P* < 0.001) after adjusting for gender, age, race, household poverty-to-income ratio, education, and marital status in Model 2. Smoking status, drinking status, exercise equivalents, hypertension, and diabetes were added to Model 3, and the odds ratio (OR) was 5.00 (95% CI, 3.75–6.67; *P* < 0.001).

**Table 3:** Logistic regression results showed an association between the Ln UHR index and BMI-defined obesity in older adults.

	**Model 1, OR (95% CI)**	**Model 2, OR (95% CI)**	**Model 3, OR (95% CI)**
LnUHR	4.25 (3.39–5.32)	6.56 (4.92–8.75)	5.00 (3.75–6.67)
	< 0.001	< 0.001	< 0.001
**LnUHR (quartiles)**			
*Q*1	Ref	Ref	Ref
*Q*2	2.31 (1.54–3.47)	2.74 (1.84–4.10)	2.60 (1.73–3.90)
	< 0.001	< 0.001	< 0.001
*Q*3	3.67 (2.47–5.45)	4.99 (3.40–7.32)	4.17 (2.83–6.15)
	< 0.001	< 0.001	< 0.001
*Q*4	5.23 (3.71–7.38)	8.34 (5.54–12.57)	6.13 (4.01–9.39)
	< 0.001	< 0.001	< 0.001
*P* for trend	< 0.001	< 0.001	< 0.001

Model 1: Unadjusted.

Model 2: adjusted for age, gender, race, education, poverty-to-income ratio, marital status.

Model 3: Adds smoking status, drinking status, exercise equivalency, hypertension, and diabetes to Model 2.

**Table 4:** Logistic regression results showed an association between the Ln UHR index and obesity defined by WHtR in older adults.

	**Model 1, OR (95% CI)**	**Model 2, OR (95% CI)**	**Model 3, OR (95% CI)**
LnUHR	10.75 (6.58–17.57)	18.33 (10.17–33.06)	15.67 (8.92–27.55)
	< 0.001	< 0.001	< 0.001
**LnUHR (quartiles)**			
*Q*1	Ref	Ref	Ref
*Q*2	2.78 (1.51–5.13)	3.41 (1.86–6.25)	3.17 (1.72–5.84)
	0.002	< 0.001	< 0.001
*Q*3	6.82 (3.44−13.50)	8.91 (4.35–18.26)	7.11 (3.45−14.63)
	< 0.001	< 0.001	< 0.001
*Q*4	20.64 (8.87–48.02)	30.72 (12.65−74.61)	20.21 (8.33–49.02)
	< 0.001	< 0.001	< 0.001
*P* for trend	< 0.001	< 0.001	< 0.001

Model 1: Unadjusted.

Model 2: adjusted for age, gender, race, education, poverty-to-income ratio, marital status.

Model 3: Adds smoking status, drinking status, exercise equivalency, hypertension, and diabetes to Model 2.

For UHR vs. obesity in older adults defined by WHtR, the odds ratio (OR) was 10.75 (95% CI,6.58–17.57; *P* < 0.001) without any adjustment for variables, as shown in Model 1. After adjusting for gender, age, race/ethnicity, household poverty-to-income ratio, education, and marital status in Model 2, the odds ratio (OR) was 18.33 (95% CI, 10.17–33.06; *P* < 0.001). Smoking status, drinking status, exercise equivalents, hypertension, and diabetes were added to Model 3, and the odds ratio (OR) was 15.67 (95% CI, 8.92–27.55; *P* < 0.001). This trend suggests that UHR is an independent risk factor for obesity in older adults and that its risk effect remains robust even after full correction for potential confounding variables.

### Nonlinear associations and threshold effect

**Figure 2:**
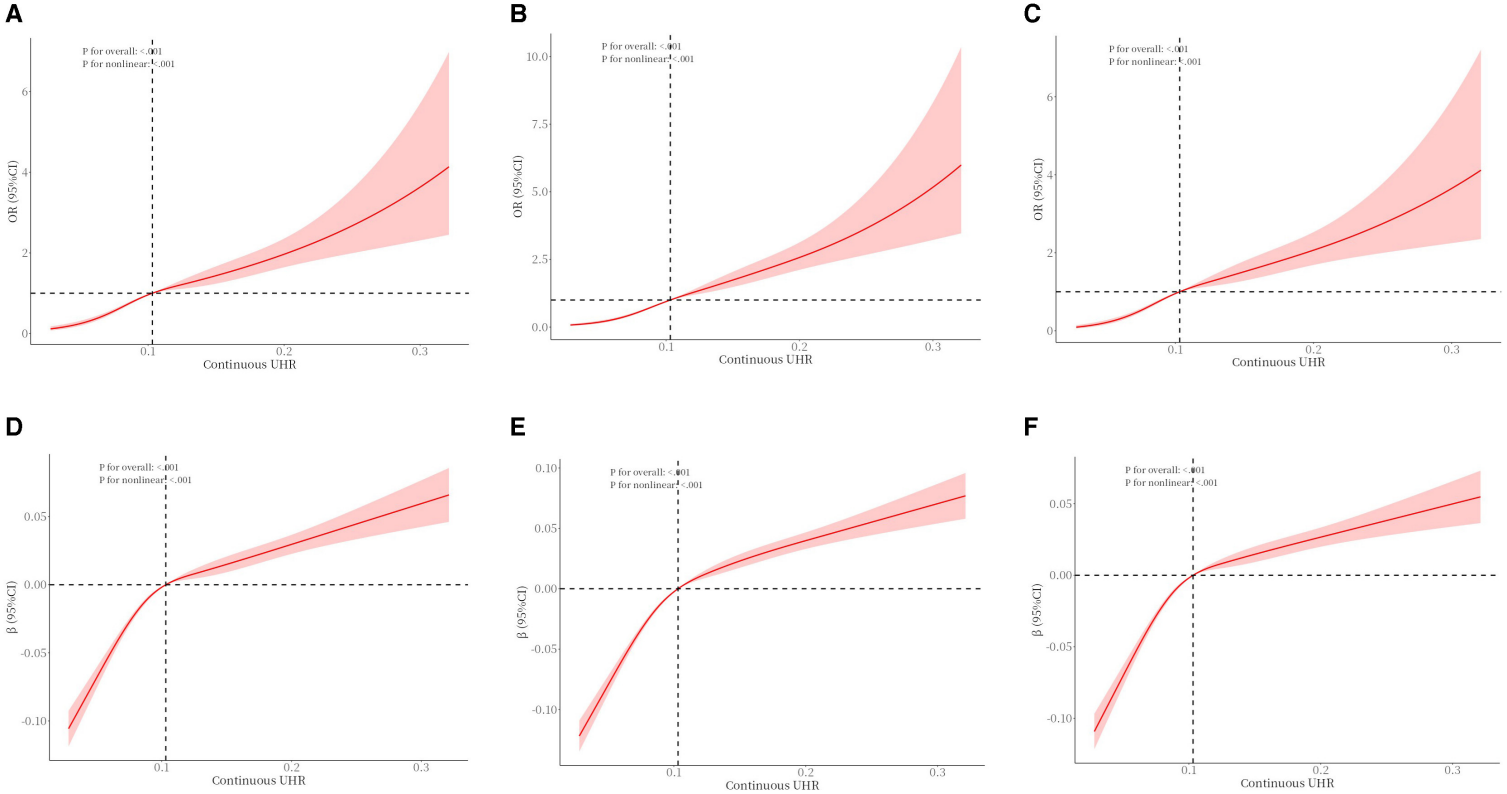
The association between UHR and obesity (defined by BMI) **(A–C)** in older adults and the association between UHR and WHtR **(D–F)** in older adults. **(A)** unadjusted. **(B)** Adjusted for age, gender, race, education, poverty-to-income ratio, and marital status. **(C)** Adds smoking status, drinking status, exercise equivalency, hypertension, and diabetes to Model B. **(D)** unadjusted. **(E)** Adjusted for age, gender, race, education, poverty-to-income ratio, and marital status. **(F)** Adds smoking status, drinking status, exercise equivalency, hypertension, and diabetes to Model E.

To examine the nonlinear dose-response relationship between the UHR index and obesity prevalence in older adults, restricted cubic spline analyses were implemented across three adjusted models (Figures 2A–F) As illustrated in the figure, nonlinear associations were consistently observed in all models, demonstrating a direct proportional relationship where elevated UHR indices corresponded with progressively higher obesity risks in older adults. The analytical framework employed restricted cubic splines with full covariate adjustment across models. Odds ratios (ORs) for obesity outcomes are presented as solid crimson trajectories and 95% confidence intervals depicted through semi-transparent red shading. Segmented regression models were used to calculate the threshold effect for each interval. In the relationship between UHR and obesity, as defined by BMI, the inflection point of the UHR index was −2.485. A significant positive correlation was observed when the UHR index was lower than −2.485 (OR = 14.96, 95% CI: 6.73, 33.25); a significant positive correlation was observed when the UHR index exceeded −2.485 (OR = 3.23, 95% CI: 2.40, 4.34). In the relationship between UHR and WHtR, the inflection point of the UHR index was −2.503, and when the UHR index was below −2.503, a significant positive correlation was observed (OR = 0.11, 95% CI: 0.09, 0.12); when the UHR index exceeded −2.503, a significant positive correlation was observed (OR = 0.06, 95% CI: 0.04–0.07) (Tables 5, 6).

**Table 5:** Nonlinearity addressed through a two-piecewise linear model (obesity defined by BMI).

**Outcome**	**Effect**	***P***
Model 1 Fitting model by standard linear regression	4.89 (4.01–5.96)	< 0.001
**Model 2 Fitting model by two-piecewise linear regression**		
Inflection point	−2.485	
< -2.485	14.96 (6.73–33.25)	< 0.001
≥-2.485	3.23 (2.40–4.34)	< 0.001
*P* for likelihood test		< 0.001

CI, confidence interval; OR, odds ratio, UHR serum uric acid/high-density lipoprotein cholesterol ratio.

**Table 6:** Nonlinearity addressed through two-piecewise linear model (WHtR).

**Outcome**	**Effect**	***P***
Model 1 Fitting model by standard linear regression	0.07 (0.07–0.08)	< 0.001
**Model 2 Fitting model by two-piecewise linear regression**		
Inflection point	−2.503	
< -2.503	0.11 (0.09–0.12)	< 0.001
≥-2.503	0.06 (0.04–0.07)	< 0.001
P for likelihood test		< 0.001

CI, confidence interval; OR, odds ratio; UHR serum uric acid/high-density lipoprotein cholesterol ratio.

### ROC curve

**Figure 3:**
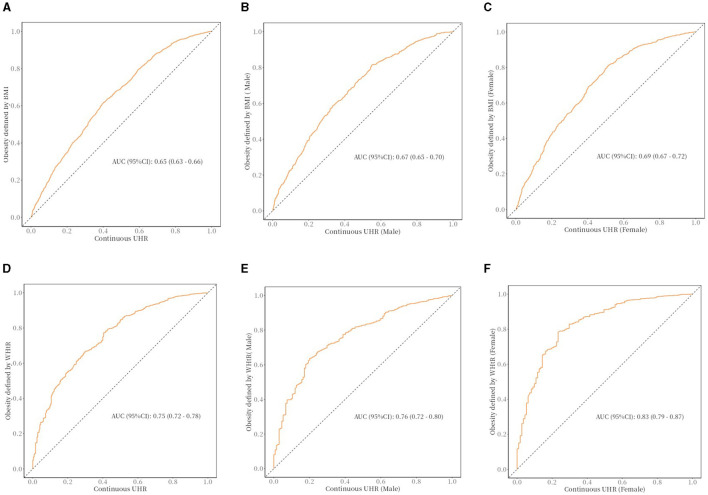
The ROC curve between UHR and obesity in older adults. **(A)** The ROC curve between UHR and obesity (defined by BMI) in older adults; cut off: 0.108. **(B)** The ROC curve between UHR and obesity (defined by BMI) in older adults (male); cut off: 0.109. **(C)** The ROC curve between UHR and obesity (defined by BMI) in older adults (female); cut off: 0.076. **(D)** The ROC curve between UHR and obesity (defined by WHtR) in older adults; cut off: 0.09. **(E)** The ROC curve between UHR and obesity (defined by WHtR) in older adults (male); cut off: 0.113. **(F)** The ROC curve between UHR and obesity (defined by WHtR) in older adults (female); cut off: 0.067.

A receiver operating characteristic curve (ROC) analysis was used to assess the predictive performance of UHR about obesity risk in older adults (Figure 3). The area under the curve (AUC) indicates the predictive value of the index; a larger AUC indicates a better predictive value, while a smaller AUC indicates a lower predictive value. As shown, the ROC curve demonstrates the predictive power of the UHR for obesity risk in older adults. In the receiver operating characteristic analysis, the UHR predicted the AUC for obesity as defined by BMI (95% CI): 0.65 (0.63–0.66). UHR predicted AUC for obesity as defined by body mass index (BMI) in older men (95% CI): 0.67 (0.65–0.70), UHR predicted AUC for obesity as defined by body mass index (BMI) in older women (95% CI): 0.69 (0.67- 0.72). The UHR predicted the AUC for predicting obesity as defined by WHtR (95% CI): 0.75 (0.72–0.78). AUC (95% CI) for obesity in older men as defined by UHR predicted body mass index (WHtR): 0.76 (0.72–0.80), AUC (95% CI) for obesity in older women as defined by UHR predictive body mass index (WHtR): 0.83 (0.79–0.87).

### Subgroup analysis

In this study, subgroup analyses and interaction tests were conducted to assess differences in the association between UHR and obesity defined by body mass index (BMI) or obesity defined by WHtR in older adults across subgroups to investigate the heterogeneity of this association. As shown in Tables 7, 8, none of the interaction *P*-values for the subgroups reached the critical value of statistical significance (all interaction *P*-values were more significant than 0.05), indicating that demographic characteristics, behavioral patterns, and health status variables did not have a significant effect on the positive association between UHR and obesity, and that, based on the results of the interaction test, the possibility of multiplicity in the hypothesis test leading to false positives was effectively controlled, and therefore no Bonferroni correction. The above results indicate a high degree of consistency in the positive association between UHR and obesity in older adults in subgroups with different demographic characteristics, behavioral patterns, and health status, which supports the robustness and generalizability of the relationship.

**Table 7:** Subgroup analysis for the association between UHR and obesity defined by BMI in older adults.

**Variables**	***n* (%)**	**OR (95%CI)**	***P***	***P* for interaction**
All patients	3,822 (100.00)	4.25 (3.39–5.32)	**< 0.001**	
Age				0.174
60–70	2,071 (54.19)	5.04 (3.49−7.26)	**< 0.001**	
70–80	1,133 (29.64)	3.66 (2.83–4.73)	**< 0.001**	
≥80	618 (16.17)	3.06 (1.84–5.08)	**< 0.001**	
Gender				0.222
Male	1,900 (49.71)	7.84 (5.23–11.76)	**< 0.001**	
Female	1,922 (50.29)	5.51 (3.70–8.22)	**< 0.001**	
Education				0.238
< 9th Grade	548 (14.34)	2.17 (1.37–3.46)	**0.002**	
9–11th Grade	487 (12.74)	5.47 (3.05–9.82)	**< 0.001**	
High School Grad/GED or Equivalent	888 (23.23)	3.30 (1.98–5.49)	**< 0.001**	
Some College or AA degree	1,064 (27.84)	4.72 (3.13–7.12)	**< 0.001**	
College Graduate or above	835 (21.85)	4.81 (3.17–7.29)	**< 0.001**	
Married				0.190
Yes	1,703 (44.56)	3.59 (2.67–4.82)	**< 0.001**	
No	2,119 (55.44)	4.94 (3.44–7.09)	**< 0.001**	
Smoking				0.809
Yes	1,934 (50.60)	4.30 (3.21–5.76)	**< 0.001**	
No	1,888 (49.40)	4.09 (2.97–5.65)	**< 0.001**	
Alcohol				0.120
Yes	2,546 (66.61)	4.81 (3.59–6.46)	**< 0.001**	
No	1,276 (33.39)	3.16 (2.07–4.80)	**< 0.001**	
Physical activity				0.673
High physical activity	1,905 (49.84)	3.89 (2.83–5.35)	**< 0.001**	
Low physical activity	1,917 (50.16)	4.36 (2.97–6.41)	**< 0.001**	
Hypertension				0.531
Yes	2,686 (70.28)	4.27 (3.20–5.69)	**< 0.001**	
No	1,136 (29.72)	3.44 (2.01–5.90)	**< 0.001**	
Diabetes				0.167
Yes	1,354 (35.43)	2.66 (1.87–3.79)	**< 0.001**	
No	2,468 (64.57)	4.05 (2.82–5.80)	**< 0.001**	

Bolded *p*-values: statistically significant.

**Table 8:** Subgroup analysis for the association between UHR and obesity defined by WHtR in older adults.

**Variables**	***n* (%)**	**OR (95%CI)**	***P***	***P* for interaction**
All patients	3,822 (100.00)	10.75 (6.58–17.57)	**< 0.001**	
Age				0.702
60-70	2,071 (54.19)	9.74 (5.41–17.52)	**< 0.001**	
70-80	1,133 (29.64)	13.61 (5.93–31.23)	**< 0.001**	
≥80	618 (16.17)	10.83 (4.34–27.00)	**< 0.001**	
Gender				0.073
Male	1,900 (49.71)	12.16 (5.94–24.89)	**< 0.001**	
Female	1,922 (50.29)	27.63(11.32–67.48)	**< 0.001**	
Education				0.053
Less Than 9th Grade	548 (14.34)	2.43 (0.76–7.81)	0.142	
9–11th Grade	487 (12.74)	8.71 (2.15–35.32)	**0.004**	
High School Grad/GED or Equivalent	888 (23.23)	3.37 (1.30–8.74)	**0.016**	
Some College or AA degree	1,064 (27.84)	20.00 (7.81–51.18)	**< 0.001**	
College Graduate or above	835 (21.85)	11.64 (5.54–24.48)	**< 0.001**	
Married				0.620
Yes	2,119 (55.44)	9.80 (5.02–19.15)	**< 0.001**	
No	1,703 (44.56)	12.70 (6.05–26.68)	**< 0.001**	
Smoking				0.141
Yes	1,934 (50.60)	7.44 (3.90–14.20)	**< 0.001**	
No	1,888 (49.40)	15.18 (7.70–29.92)	**< 0.001**	
Alcohol				0.801
Yes	2,546 (66.61)	10.86 (6.28–18.80)	**< 0.001**	
No	1,276 (33.39)	9.65 (4.20–22.16)	**< 0.001**	
Physical activity				0.957
High physical activity	1,905 (49.84)	10.62 (3.92–28.76)	**< 0.001**	
Low physical activity	1,917 (50.16)	10.29 (5.62–18.84)	**< 0.001**	
Hypertension				0.518
Yes	2,686 (70.28)	8.57 (4.27–17.17)	**< 0.001**	
No	1,136 (29.72)	11.85 (6.03–23.32)	**< 0.001**	
Diabetes				0.386
Yes	1,354 (35.43)	5.85 (2.32–14.76)	**< 0.001**	
No	2,468 (64.57)	9.59 (5.47–16.80)	**< 0.001**	

Bolded *p*-values: statistically significant.

## Discussion

Using NHANES data from 2011 to 2016, this study examined the relationship between UHR and obesity in older adults. After a series of inclusion and exclusion, this study included as many as 3,822 cases. The data analysis revealed a positive relation between UHR and the risk of obesity in older adults, and this correlation was still evident after taking potential confounding factors into account. When the UHR was treated as a categorical variable, the risk of obesity defined by BMI in (Q4) was 6.13 times higher than that in (Q1). The risk of obesity defined by WHtR in (Q4) was 20.21 times higher than that in (Q1). Another noteworthy finding was a significant nonlinear dose-response relation between UHR and obesity in older adults. According to the ROC curve, the UHR can be an important tool for identifying older women at high risk for obesity. The analytical results provided evidence of a statistically significant positive relation between UHR and obesity in the geriatric population.

In older adults, BMI may underestimate obesity-related health risks (inability to differentiate fat distribution), resulting in a weaker predictive power of the UHR index. WHtR may be more appropriate for the older adults, as it is more sensitive to central obesity (independent of age-related muscle loss). The UHR index is more valuable in identifying metabolic risk-associated obesity (i.e., visceral adiposity), suggesting its potential as a complementary clinical tool. The UHR index could serve as a crucial therapeutic intervention target and a significant predictive factor in developing obesity prevention strategies for older adult populations. The consistency of the UHR-obesity association across heterogeneous population strata (varying in demographic composition, lifestyle patterns, and clinical status) was substantiated through rigorous subgroup analyses and interaction evaluations, confirming the epidemiological robustness of this metabolic relationship. This result confirms our initial hypothesis and emphasizes the important relationship between UHR and obesity in older adults. To formulate practical therapeutic approaches for older adults, it is crucial to thoroughly comprehend the relationship between UHR and adiposity-related health conditions in aging individuals. The uric acid and high-density lipoprotein cholesterol levels can be regulated by interventions such as a healthy diet, exercise, smoking, and drinking cessation. A series of measures may help reduce the index of UHR in older adults, reducing the risk of obesity.

Understanding the complex relationship between UA and HDL is essential for assessing health outcomes. Serving as a composite marker, its association with a range of metabolic disorders, such as non-alcoholic fatty liver disease (NAFLD) (34) and hypertension (33), has been established. The UHR indicates the dynamic equilibrium between the lipid-promoting attributes of UA and the fat-breaking function of HDL-C (42). The interaction between inflammatory and oxidative mechanisms in disease progression is revealed. Adipose tissue hypertrophy and hyperplasia in obesity are associated with hypoxic microenvironments that induce adipocyte apoptosis and necrosis, subsequently generating excessive reactive oxygen species (ROS) and activating oxidative stress pathways (43). In addition, the adipose tissue of obese people secrets a large number of pro-inflammatory factors, such as tumor necrosis factor-α (TNF-α) and interleukin-6 (IL-6), which not only cause inflammation in adipose tissue but also spread to the whole body through the systemic circulation (44). At the same time, macrophages in adipose tissue are also activated, further aggravating inflammation (45). Our study found a nonlinear relationship between uric acid to high-density lipoprotein cholesterol ratio (UHR) and obesity risk in older adults, whereas previous studies reported an inverted U-shaped relationship between uric acid and obesity. The ambivalent biological role of uric acid (UA) manifests as a concentration-dependent biphasic impact on adipogenesis regulation, demonstrating both protective and pathogenic potentials within obesity-related metabolic pathways. At physiological concentrations, UA reduces the generation of reactive oxygen species (ROS) by inhibiting NADPH oxidase activity and protecting mitochondrial function in adipocytes. However, chronic hyperuricemia can activate NLRP3 inflammasome, promote macrophage infiltration and IL-1β release in adipose tissue, and induce adipocyte hypertrophy and fibrosis (46). HDL-C and its major apolipoprotein component, ApoA-I, exert significant anti-inflammatory and organ-protective effects by scavenging lipopolysaccharides (LPS), suppressing pro-inflammatory cytokine production, and improving endothelial function (47). Thus HDL-C reduces the gene transcription of pro-inflammatory factors such as TNF-α, IL-6, and IL-8 (48). The role of uric acid and high-density lipoprotein (HDL) cholesterol in the pathogenesis of obesity and obesity-associated diseases is well supported by the available evidence (49, 50). Elevated UA levels, commonly seen in obese individuals, may exacerbate adipose tissue inflammation through xanthine oxidase-driven oxidative stress and impaired insulin signal (51).

High-density lipoprotein cholesterol (HDL-C) plays a protective role against obesity by improving adipose tissue function, modulating the secretion of adipokines such as adiponectin and leptin, and exerting anti-inflammatory and antioxidant effects (52). The superior predictive ability of the uric acid to HDL-C ratio (UHR) for obesity may be partly attributed to its reflection of uric acid-driven lipogenesis. Uric acid has been shown to induce mitochondrial oxidative stress in hepatocytes, inhibit aconitase activity in the tricarboxylic acid cycle, promote cytoplasmic citrate accumulation, and activate key lipogenic enzymes such as ATP-citrate lyase and fatty acid synthase, thereby enhancing de novo lipid synthesis (53). HDL-C-mediated lipid clearance: HDL-C enhances reverse cholesterol transport through the ABCA1/G1 pathway and inhibits ectopic lipid deposition by up-regulating adipose triglyceride lipase (ATGL) (54, 55).

This research contributes novel insights by (1) establishing UHR as a feasible biomarker for geriatric obesity risk assessment, (2) elucidating demographic-specific patterns of UHR-obesity associations, and (3) providing accurate evidence for clinical interpretation. These findings advance our pathophysiological understanding of metabolic dysregulation in aging populations while proposing practical tools for targeted screening and preventive intervention development. The utilization of the UHR index as a multidimensional biomarker has been demonstrated to establish innovative frameworks for enhancing diagnostic paradigms in geriatric adiposity assessment, and the interaction between oxidative and inflammatory mechanisms sheds light on the biological basis of this disease. So, the UHR holds promise in the clinical diagnosis, therapeutic surveillance, and prognostic assessment of obesity in older adults. This study demonstrates notable strengths, including the novel integration of UA and HDL-C as a composite biomarker. Previous studies have examined these biomarkers individually and may have overlooked their dynamic interactions in metabolic homeostasis. Our findings extend current knowledge by demonstrating that the UHR index (integrating UA and HDL-C) is associated with obesity metrics (e.g., waist circumference, visceral fat area) more than either component system alone. Compared to single metrics, UHR can identify older subclinical obese populations with a propensity for abnormal visceral fat accumulation 3–5 years earlier. This furnishes a valuable tool for examining its association with obesity in the aged. UHR has more obvious advantages than other metabolic markers: UHR can reflect the advantages of uric acid metabolism and lipid metabolism disorders at the same time, especially in obesity-related metabolic syndrome, which has a high value of application. However, metabolic heterogeneity exists in older adults obese population, and some patients may show “metabolically healthy obesity” (no apparent metabolic abnormality), while UHR mainly relies on uric acid and HDL-C and is unable to comprehensively assess other metabolic disorders, such as insulin resistance and inflammatory status. In terms of the study population, Analytical procedures were conducted using de-identified demographic and health metrics from the 2011–2016 cycle of NHANES to ensure the accuracy of the study as well as the breadth of the population. Based on this, the study weighted the data. It used multi-model logistic regression, restricted cubic spline curves, threshold effect, and subgroup analyses to control for various types of confounding factors, thus making the results more accurate. This study demonstrates that elevated UHR index levels correlate with increased obesity prevalence among older adults, carrying significant population health ramifications for combating and detecting obesity in this demographic.

Nevertheless, our research has certain constraints. The fact that this investigation is cross-sectional comes first in our analysis. This was able to determine associations between exposure factors and outcomes, but it could not determine their causal relationship. Therefore, it is necessary to perform Mendelian randomization, for example, to confirm causality and to explore tissue-specific effects through animal models. As a second point, the self-reported confounders can be easily influenced by biased recall. In addition, we might have failed to identify some potentially confounding variables; the possibility of bias remains. Secondly, the research concentrated on individuals aged 60 and above; as a result, Caution is warranted when extrapolating these findings to individuals under 60. Furthermore, the issues of whether UHR can be combined with other indicators for predicting obesity in older adults and the timing of UHR measurements need to be addressed. Subsequent experiments will aid in comprehending the potential mechanisms underlying the association between UHR and obesity.

## Conclusion

UHR is independently associated with obesity in older adults. UHR can be used as a supportive indicator for obesity risk stratification, but further validation in prospective cohorts is needed. Interventions targeting UHR modulation may contribute to developing targeted obesity prevention strategies in aging populations.

## Data Availability

The original contributions presented in the study are included in the article/supplementary material, further inquiries can be directed to the corresponding author.
